# Supplementary material for: The Incremental Cluster Threshold-Free Cluster Enhancement Algorithm for Functional Connectivity Analysis
Source: bioRxiv. 2026 Apr 16:2026.04.06.716826. Preprint. [Version 2] doi: 10.64898/2026.04.06.716826 (PMC13104858; doi:10.64898/2026.04.06.716826)
Supplement: Supplement 1 [file NIHPP2026.04.06.716826v2-supplement-1.pdf]

bioRxiv preprint doi: https://doi.org/10.64898/2026.04.06.716826; this version posted April 16, 2026. The copyright holder for this preprint (which was not certified by peer review) is the author/funder, who has granted bioRxiv a license to display the preprint in perpetuity. It is made available under aCC-BY 4.0 International license.

7 TFCE and IC-TFCE Equality

Let $G = (N, E, W)$ be an undirected weighted graph generated from the calculated t-statistics from a GLM fit, with N being the set of nodes, E being the set of edges, and W a function from $E \to \mathbb{R}$ that returns a t-statistic associated with an edge $e \in E$.

We assume the existence of at least one positively weighted edge in G:

$$\exists\, e \in E \quad \text{such that} \quad W(e) > 0.$$

and that the number of edges is finite:

$$|E| \leq B_e \tag{11}$$

with $B_e \in \mathbb{N}_{>0}$ and $|E|$ the number of edges.

Furthermore, we assume the function W is bounded, so it maps all finite number of edges to finite values. Therefore, there exists a $B_w \in \mathbb{R}$ such that for all $e \in E$:

$$W(e) \leq B_w \tag{12}$$

As a final assumption, we assume the graph is undirected: if $(n_i, n_j) \in E$, then $(n_j, n_i) \in E$ and $W(n_i, n_j) = W(n_j, n_i)$. Consequently, paths in G are reversible. If there exists a path from node n_a to node n_b implies the reverse path from n_b to n_a is also in the graph. The assumption holds for the symmetric functional connectivity graphs. For simplification, this assumption will remain implicit for the extent of this proof and paths will be considered symmetric.

Let $\delta t > 0, \delta t \in \mathbb{R}$ be the integration step size of the TFCE algorithm.

The TFCE algorithm computes the following: the subgraphs given a cut off threshold and the clusters from that subgraph. Finally, with the cluster sizes, the TFCE integral is calculated. We start by defining the subgraphs calculated by the TFCE and then it's clusters.

TFCE subgraphs: Let $k \in \mathbb{N}$. The sequence of subgraphs $(G_k)_{k \in \mathbb{N}}$ with $G_k = (N, E_k, W)$ where E_k is a subset of edges such that

$$E_k = \{e \in E \mid W(e) \geq k \cdot \delta t\} \tag{13}$$

is the sequence of subgraphs calculated by the TFCE algorithm.

TFCE clusters: The clusters for the TFCE are the maximum connected induced subgraphs (MCISs) of the subgraph $G_k = (N, E_k, W)$. We now define a maximum connected induced subgraph.

If $G'_k = (N'_k, E'_k, W)$ is an MCIS from $G_k = (N, E_k, W)$, it is:

- For any two nodes N'_k, there exists a path between them using only edges in E'_k,
- No additional node from $N \setminus N'_k$ can be added to N'_k without violating this property (i.e. the number of nodes is maximal).
- E'_k contains all edges from E_k that connect pairs of nodes in N'_k (i.e., $E' = \{e_{i,j} \in E' \mid n_i, n_j \in N'\}$) (i.e. the number of edges is maximal).

To prove the IC-TFCE produces equal results, we need to define both the subgraphs and clusters generated by the IC-TFCE and then show that they are equal to the ones produced by the TFCE. We will start by defining the subgraphs generated by the IC-TFCE and show they are equal. Finally, we will do the same for the clusters.

IC-TFCE Subgraphs: Now, we define the subgraphs generated by the IC-TFCE implementation. We start with some set definitions.

Let $\mathcal{T} = (T_k)_{k \in \mathbb{N}}$ and $\mathcal{H} = (H_k)_{k \in \mathbb{N}}$ be sequences of sets indexed by $k \in \mathbb{N}$.

Let each $T_k \in \mathcal{T}$ be the set of edges defined as follows:

$$T_k = \{e \in E \mid k \cdot \delta t \leq W(e) < (k+1) \cdot \delta t\} \tag{14}$$

Further, let H_k be the sets defined at such:

$$H_k = \bigcup_{i \geq k, i \in \mathbb{N}} T_i \tag{15}$$

bioRxiv preprint doi: https://doi.org/10.64898/2026.04.06.716826; this version posted April 16, 2026. The copyright holder for this preprint (which was not certified by peer review) is the author/funder, who has granted bioRxiv a license to display the preprint in perpetuity. It is made available under aCC-BY 4.0 International license.

The sequence of subgraphs generated by the IC-TFCE algorithm $(I_k)_{k\in\mathbb{N}}$ are the subgraphs with $I_k = (N, H_k, W)$.

We first prove a Lemma that will be used by most induction steps in this proof. It is used to establish the base cases.

Lemma 7.1. *There exists a $K \in \mathbb{N}$ such that $E_k = H_k = \emptyset$ for $k \geq K$*

Proof. Let $w_{max} = max_{e\in E} W(e)$. The maximum is well defined because the graphs are finite and the function W is bounded. Let $K \in \mathbb{N}$ be the lowest number such that $K \cdot \delta t > w_{max}$.

From the definition of E_k, one has that

$$E_k = \emptyset \quad \text{for} \quad k \geq K \tag{16}$$

Furthermore, for all e, it is $W(e) < K\delta t$. Therefore, from the definition of T_k, one has that $T_k = \emptyset$ for $k \geq K$. It follows from the definition of H_k that $H_k = \emptyset$

It is $E_k = H_k = \emptyset$ for $k \geq K$ and the proof is concluded. □

Lemma 7.2. *For all $k \in \mathbb{N}$, we have $E_k = H_k$.*

Proof. To prove this, one can show that assuming an element belongs to E_k implies it must be in H_k, and vice-versa (i.e. mutual inclusion).

- **Let** $e \in H_k$: Since $e \in H_k$, there exists a T_i with $i \geq k$ such that $W(e) \in [i \cdot \delta t, (i+1) \cdot \delta t)$, therefore, we can conclude $W(e) \geq i \cdot \delta t$ and consequently $e \in E_k$.
- **Let** $e \in E_k$: Since $e \in E_k$, one has that $W(e) \geq k \cdot \delta t$. From Lemma 7.1, one can conclude $W(e) \in [k \cdot \delta t, K\delta t)$. Therefore, since the sets $\{[i \cdot \delta t, (i+1) \cdot \delta t)\}_{\{i\in\mathbb{N}|i\geq k, i\leq K\}}$ form a disjoint partition of $[k \cdot \delta t, K\delta t)$, there exists an index $i \in \mathbb{N}$ such that $W(e) \in [i \cdot \delta t, (i+1) \cdot \delta t)$, and $e \in T_i$ with $i \geq k$. Since $e \in T_i, i \geq k$, one has that $e \in H_k$.

It follows by subset inclusion that $H_k = E_k$. □

Corollary 7.3.

$$I_k = G_k \quad \textit{for all } k \in \mathbb{N} \tag{17}$$

Proof. This is a direct consequence of Lemma 7.2 that establishes an equality between edges. From the definition of I_k, it has the same nodes and the same weight function for edges as G_k. Therefore, the subgraphs are equal. □

Now all that is left to prove is that the IC-TFCE truly computes the correct clusters at each iteration. We start by defining the clusters outputted by the algorithm and then show they are indeed maximum connected induced subgraphs.

Definition 7.4 (IC-TFCE Cluster Construction)**.** Let $\mathcal{C}_k$ be the set of clusters produced by the IC-TFCE from the subgraph G_k, indexed by nodes $x \in N$:

$$c_k^x = (N_k^x, E_k^x, W) \in \mathcal{C}_k$$

constructed by backwards induction from K to 0 as follows:

- **Base case**: $c_K^x = (\{n_x\}, \emptyset, W)$, where n_x is a node in N indexed by $x \in \mathbb{N}$, and $K \in \mathbb{N}$ is as defined in Lemma 7.1.
- **Inductive step**: The cluster $c_{k-1}^x = (N_{k-1}^x, E_{k-1}^x, W)$ is defined as:
  - A node n is in N_{k-1}^x if there exists $m \in \mathbb{N}$ and cluster indices $y_1, \ldots, y_m \in N$ such that:
    1. $y_1 = x$
    2. $n \in N_k^{y_m}$
    3. For each $i \in \{1, \ldots, m-1\}$, there exists an edge $e \in T_{k-1}$ with one endpoint in $N_k^{y_i}$ and the other in $N_k^{y_{i+1}}$
  - An edge e is in E_{k-1}^x if both endpoints of e are in N_{k-1}^x and $e \in H_{k-1}$

bioRxiv preprint doi: https://doi.org/10.64898/2026.04.06.716826; this version posted April 16, 2026. The copyright holder for this preprint (which was not certified by peer review) is the author/funder, who has granted bioRxiv a license to display the preprint in perpetuity. It is made available under aCC-BY 4.0 International license.

Note that when clusters c^x_{k+1} and c^z_{k+1} merge at step k, we have:

$$c^x_k = c^z_k \tag{18}$$

Furthermore, note that $m = 1$ is permitted, in which case the condition is satisfied by individual nodes with a path with no necessary edges. From this, we conclude $N^x_{k+1} \subseteq N^x_k$.

The proof strategy now is to prove the clusters produced by the IC-TFCE are MCIS. As the MCIS from a subgraph G_k are unique and the TFCE computes the MCIS of subgraphs G_k, if the IC-TFCE computes the MCIS of G_k, they will be consequently equal from their uniqueness.

Lemma 7.5. *The clusters c^x_k constructed by the IC-TFCE are maximally connected induced subgraphs*

Proof. To prove the nodes and edges are maximal and the graph is connected, one can use reverse induction from K to 0. If those three requirements are satisfied, one has that the cluster is a MCIS by the definition of MCIS.

Let c^x_k be a cluster generated by the IC-TFCE algorithm.

Base case: From Lemma 7.1, one has that the set of edges is empty for the graph G_K. Therefore, as the clusters are initially defined containing the nodes alone, the clusters in $\mathcal{C}_K$ are indeed maximum connected induced subgraphs.

Induction Step:

- **Nodes are Maximal**: Assume by contradiction that the number of nodes is not maximal: there exists a node $n_1 \in N^x_{k-1}$ with an edge $e = (n_1, n_2) \in E_{k-1}$ such that $n_2 \notin N^x_{k-1}$.

  Since $e \in E_{k-1} = H_{k-1}$ (Lemma 7.2) and $E_K = \emptyset$, there exists some $K > i \geq k-1$ such that $e \in T_i$.

  At threshold i, let c^z_{i+1} be the cluster containing n_1 and let c^w_{i+1} be the cluster containing n_2. Since $e = (n_1, n_2) \in T_i$, the edge e is a bridging edge between c^z_{i+1} and c^w_{i+1} at step i. This implies $n_1 \in N^z_i$ and $n_2 \in N^z_i$ and they are both in the same cluster (indexed with z). By cluster construction, one has that $n_1, n_2 \in N^z_{k-1}$ as $N^z_i \subseteq N^z_{k-1}$. Since $n_1 \in N^x_{k-1}$, from the definition in Equation (18), one has that $N^x_{k-1} = N^z_{k-1}$ and that $n_2 \in N^x_{k-1}$ a contradiction to the assumption that $n_2 \notin N^x_{k-1}$.

  It follows that the number of nodes is maximal.

- **Edges Are Maximal**: Let us assume by contradiction that the number of edges is not maximal: there exists an edge e from $n_1 \in N^x_{k-1}$ to $n_2 \in N^x_{k-1}$ such that $e \notin E^x_{k-1}$. To avoid a contradiction with the algorithms definition, one must have $e \notin H_{k-1}$. However, from Lemma 7.2, $H_{k-1} = E_{k-1}$, so if $e \notin H_{k-1}$, it is not in the edge E_{k-1} set of the subgraph G_{k-1}. Since the cluster is from the subgraph G_{k-1} at iteration $k-1$ within the set of edges E_{k-1}, this is a contradiction and no such e is outside of this scope of edges, and consequently, the number of edges is maximal.

- **Graph is connected**: Let two nodes in $n_1, n_2 \in N^x_{k-1}$. From the definition of the set of nodes for each cluster in iteration of the algorithm, for n_1 to be in N^x_{k-1}, there exists a node $n_3 \in N^x_k$ (can be n_1) that is connected to n_1 through a path of bridging edges from the previous iteration step. Again, repeating the same argument, from the definition of the algorithm, there exists a node $n_4 \in N^x_k$, such that there is a path from n_2 to n_4. Since, one has that $n_3 \in N^x_k$ and $n_4 \in N^x_k$, there exists a path from n_3 to n_4 as N^x_k as is a set of nodes from the cluster c^x_k must be a connected graph from the induction assumption.

  Therefore, the path:

  $$n_1 \to n_3 \to n_4 \to n_2 \tag{19}$$

  proves that the nodes n_1 and n_2 are connected by a path.

With this induction, one can conclude c^x_{k-1} is a MCIS and the proof is concluded.

□

From the results from Corollary 7.3, we establish that the edge allocation procedure from the IC-TFCE creates subgraphs equal to the TFCE. On Lemma 7.5, we establish that the clusters from IC-TFCE are indeed MCIS. Since its folklore that MCIS are unique for a given graph, it follows that IC-TFCE generates the same clusters as the traditional TFCE implementation.

bioRxiv preprint doi: https://doi.org/10.64898/2026.04.06.716826; this version posted April 16, 2026. The copyright holder for this preprint (which was not certified by peer review) is the author/funder, who has granted bioRxiv a license to display the preprint in perpetuity. It is made available under aCC-BY 4.0 International license.

7.1 Extension to Exact TFCE

It suffices to propose T_k such that

$$T_k = \{e \in E \,|\, w_i \leq W(e) < w_{i+1}\} \tag{20}$$

where $(w_i)_{i\in\mathbb{N}}$ is the ordered (monotonically increasing) sequence of weights for all images of $W(e)$ with $e \in E$. The conclusions and reasoning from proofs from the IC-TFCE would follow.

8 Voxel Graph Transformation

In this section, we prove that the graph-based transformation yields clusters equivalent to the traditional voxel cluster definition used in neuroimaging.

8.1 Voxel Data and Traditional Clusters

Let $\mathcal{V}$ be a finite set of voxels and let $\sim$ denote spatial adjacency (voxels neighboring each other) on $\mathcal{V}$, where we include self-adjacency (i.e., $v \sim v$ for all $v \in \mathcal{V}$). Let $T : \mathcal{V} \to \mathbb{R}$ be a function mapping each voxel to its test statistic. We refer to the triple $(\mathcal{V}, \sim, T)$ as *voxel data*.

Given a threshold $h \in \mathbb{R}$, a voxel $v_x \in \mathcal{V}$, and voxel data $\mathcal{D} = (\mathcal{V}, \sim, T)$, the thresholded cluster definition which is used by the voxel-based TFCE and cluster size inference in neuroscience:

$$\mathcal{N}_h(v_x, \mathcal{D}) = \left\{ v \in \mathcal{V} \;\middle|\; \begin{array}{l} \exists \text{ sequence } (u_0, u_1, \ldots, u_k) \text{ with } u_0 = v_x,\ u_k = v, \\ u_i \sim u_{i+1} \text{ for all } i < k, \text{ and } T(u_i) \geq h \text{ for all } i \leq k \end{array} \right\} \tag{21}$$

Intuitively this means that if you take any voxel in the cluster, you can recreate the cluster by using adjacent paths above the desired threshold.

8.2 Graph Transformation

We define the equivalent graph transformation Γ that maps voxel data to a weighted graph. Given voxel data $\mathcal{D} = (\mathcal{V}, \sim, T)$, define $\Gamma : (\mathcal{V}, \sim, T) \to G = (N, E, W)$ where:

Nodes N: Each voxel becomes a node in the graph:

$$N = \mathcal{V} \tag{22}$$

Edges E: Two nodes are connected by an edge if and only if their corresponding voxels are spatially adjacent. Recall that we include self-adjacency ($v \sim v$ for all v), so each node also has a self-loop:

$$E = \{(v_1, v_2) \in \mathcal{V} \times \mathcal{V} \mid v_1 \sim v_2\} \tag{23}$$

Weights W: The weight function maps an edge to the minimum test statistic of its two endpoints:

$$W(v_1, v_2) = \min\{T(v_1), T(v_2)\} \text{ with } v_1, v_2 \in \mathcal{V} \tag{24}$$

Note that the self-adjacency condition ensures that the edges from a node to itself $(v, v) \in E$ result in $W(v, v) = T(v)$ for each voxel.

8.3 Graph-Based Clusters

Given a threshold $h \in \mathbb{R}$, define the following subset of edges from E:

$$E_h = \{e \in E \mid W(e) \geq h\} \tag{25}$$

For a node $v_x \in N$ and voxel data $\mathcal{D}$, define the *graph cluster* $\mathcal{S}_h(v_x, \Gamma(\mathcal{D}))$ as the set of nodes reachable from v_x via edges in E_h:

$$\mathcal{S}_h(v_x, \Gamma(\mathcal{D})) = \left\{ v \in N \;\middle|\; \begin{array}{l} \exists \text{ sequence of edges } (e_1, \ldots, e_k) \subseteq E_h, \text{with } k \geq 1, \text{ forming} \\ \text{a path from } v_x \text{ to } v \end{array} \right\} \tag{26}$$

bioRxiv preprint doi: https://doi.org/10.64898/2026.04.06.716826; this version posted April 16, 2026. The copyright holder for this preprint (which was not certified by peer review) is the author/funder, who has granted bioRxiv a license to display the preprint in perpetuity. It is made available under aCC-BY 4.0 International license.

8.4 Equivalence of Cluster Definitions

Theorem 8.1. *Let $G = \Gamma(\mathcal{D})$ be the equivalent graph transformation applied to voxel data $\mathcal{D} = (\mathcal{V}, \sim, T)$. For any threshold $h \in \mathbb{R}$ and any voxel $v_x \in \mathcal{V}$:*

$$\mathcal{N}_h(v_x, \mathcal{D}) = \mathcal{S}_h(v_x, \Gamma(\mathcal{D})) \tag{27}$$

Proof. One can show that the sets are equal through mutual inclusion.

- **Let** $v \in \mathcal{S}_h(v_x, \Gamma(\mathcal{D}))$: From the definition of $\mathcal{S}_h(v_x, \Gamma(\mathcal{D}))$, there exists a path of edges in E_h from v_x to v, corresponding to a sequence of voxels $(u_0, u_1, \ldots, u_k)$ with $u_0 = v_x$ and $u_k = v$.

  For each edge (u_i, u_{i+1}) in this path, one has $W(u_i, u_{i+1}) \geq h$, which implies $\min\{T(u_i), T(u_{i+1})\} \geq h$. Consequently, for all $i \leq k$, it is $T(u_i) \geq h$.

  Since each $(u_i, u_{i+1}) \in E_h \subseteq E$, it follows from the definition of E that $u_i \sim u_{i+1}$ for all $i < k$. Considering the following statements: it is $u_0 = v_x$, $u_k = v$, and $T(u_i) \geq h$ for all $i \leq k$, the sequence $(u_0, \ldots, u_k)$ satisfies all conditions in the definition of $\mathcal{N}_h(v_x, \mathcal{D})$, so $v \in \mathcal{N}_h(v_x, \mathcal{D})$.

- **Let** $v \in \mathcal{N}_h(v_x, \mathcal{D})$: From the definition of $\mathcal{N}_h(v_x, \mathcal{D})$, there exists a sequence of adjacent voxels $(u_0, u_1, \ldots, u_k)$ with $u_0 = v_x$, $u_k = v$, and $T(u_i) \geq h$ for all $i \leq k$.

  Since $u_i \sim u_{i+1}$, the edge $(u_i, u_{i+1}) \in E$ for all $i \leq k$.

  Since one has both $T(u_i) \geq h$ and $T(u_{i+1}) \geq h$ for each consecutive pair, one has:

  $$W(u_i, u_{i+1}) = \min\{T(u_i), T(u_{i+1})\} \geq h \tag{28}$$

  From $(u_i, u_{i+1}) \in E$ and Equation (28), it follows from $(u_i, u_{i+1}) \in E_h$ for all $i \leq k$.

  It follows there exists a path in E_h from v_x to v, so $v \in \mathcal{S}_h(v_x, \Gamma(\mathcal{D}))$.

One can conclude that the sets are indeed equal.

□
